# Environmental and Cognitive Enrichment in Childhood as Protective Factors in the Adult and Aging Brain

**DOI:** 10.3389/fpsyg.2020.01814

**Published:** 2020-07-21

**Authors:** Bertrand Schoentgen, Geoffroy Gagliardi, Bénédicte Défontaines

**Affiliations:** ^1^Réseau Aloïs Pôle Enfant (Pediatric Aloïs Network), Paris, France; ^2^Réseau Aloïs (Aloïs Network), Paris, France; ^3^UPMC Univ Paris 06, Inserm, CNRS, Institut du Cerveau et de la Moelle (ICM) – Hôpital Pitié-Salpêtrière, Sorbonne Universités, Paris, France

**Keywords:** cognitive reserve, childhood, development, aging brain, lifespan, environmental enrichment

## Abstract

Some recent studies have highlighted a link between a favorable childhood environment and the strengthening of neuronal resilience against the changes that occur in natural aging neurodegenerative disease. Many works have assessed the factors – both internal and external – that can contribute to delay the phenotype of an ongoing neurodegenerative brain pathology. At the crossroads of genetic, environmental and lifestyle factors, these relationships are unified by the concept of cognitive reserve (CR). This review focuses on the protective effects of maintaining this CR through the cognitive aging process, and emphasizes the most essential time in life for the development and strengthening of this CR. The in-depth study of this research shows that early stimulation with regard to social and sensory interactions, contributes to the proper development of cognitive, affective and psychosocial capacities. Childhood thus appears to be the most active phase in the development of CR, and as such we hypothesize that this constitutes the first essential period of primary prevention of pathological aging and loss of cognitive capacities. If this hypothesis is correct, early stimulation of the environment would therefore be considered as a true primary prevention and a public health issue. The earlier identification of neurodevelopmental disorders, which can affect personal and professional development across the lifespan, could therefore have longer-term impacts and provide better protection against aging.

## Introduction

The first years of life – from birth to adolescence – are the most defining period of brain development, organization and specialization (Giedd et al., 1999; Johnson, 2001). This development is made possible by a densification of synaptic and dendritic networks as well as an increase in brain volume. In the neurodevelopmental field, research has observed child brain development through both the genetic and environmental lens (Gogtay et al., 2004; Baroncelli et al., 2010; Rao et al., 2010; Anderson et al., 2011; Huang et al., 2013). Environmental stimulations would actively participate in the development of language, motor skills, behavior and emotional management; prerequisites of greater intellectual flexibility (Hochberg et al., 2010; Murgatroyd and Spengler, 2011). Studies have advanced the idea of a link between these favorable conditions and neuronal resilience during natural aging of the brain or potential further brain damage (Stern, 2006).

On the other hand, aging is associated with new diseases affecting cognition (e.g., dementia). The WHO reports that approximately 46.8 million people currently live with dementia (Prince, 2015). This number tends to double almost every two decades (Prince et al., 2016), leading to around 135 million potential patients in 2050 (Wimo et al., 2017), a frequently increasing estimation (Prince et al., 2013). This phenomenon has become one of the greatest challenges of the 21st century, warranting regard as a public health priority, a social emergency affecting mostly developing countries. The annual cost of care for a patient diagnosed with dementia is also increasing (i.e., from $15,122 in 2010 to $17,483 in 2015), leading to a simulated world cost of $2 trillion in 2030 (Wimo et al., 2017). Taken together, these arguments constitute a worldwide health issue. Given the absence of striking effective pharmacological (Bahar-Fuchs et al., 2013; Cooper et al., 2013; Epelbaum et al., 2017) or cognitive (Sitzer et al., 2006; Olazarán et al., 2010; Woods et al., 2012; Bahar-Fuchs et al., 2013; Gates and Sachdev, 2014; Nelson and Tabet, 2015; Oltra-Cucarella et al., 2016) treatment once the pathology has begun, many studies examined modifiable risks and protective factors of dementia before the onset of clinical symptoms. One of the main factors considered, along with genetics, environment and lifestyle (e.g., diet, urban vs. rural lifestyle), seems to be CR.

Defined by Yaakov Stern in the late 1990s, the concept of CR lies on two types of results. First, at the end of the 1980s, authors found different pathological levels in patients at equivalent stage of Alzheimer’s Disease (AD) (Katzman et al., 1989; Snowdon et al., 1996). Similarly, some studies have shown a higher prevalence of dementia among individuals with lower levels of education (Riley et al., 2002). Addressing these ambiguities, CR refers to inter-individual adaptive differences to equivalent brain damage (Stern et al., 2018). These differences can be modulated by a variety of biological, environmental, psychological, educational and activity factors (Satz et al., 2011; Xu et al., 2015). Identified in studies by their proxies, these are represented by both static (e.g., intra-cranial volume) and dynamic elements (e.g., occupation). From the lifespan perspective, CR would vary beginning in, perpetually evolving over time (Katzman, 1995; Richards and Deary, 2005; Richards et al., 2013).

In this review, we want to highlight the most significant research on CR and the protective effects of these factors in adulthood. Furthermore, we will look at their effects on the cognitive aging process. Then, we will focus particularly on the source of those factors from birth and child development. We will thus discuss the concept of CR (i.e., specifically how it develops and is maintained) to help improve resilience, which would prevent brain and cognitive impairment (i.e., acquired or developmental) from childhood and throughout life. Our work examines intersections between previous findings in literature, aiming to identify CR’s origin. This theoretical work will thus make it possible to hypothesize strategies for early prevention of cognitive decline from childhood. Cognitive stimulation would have positive long-term effects, from birth and especially on aging. The issue could be a more serious consideration of public authorities on education and prevention of neurodevelopmental disorders in children, given the cost of low CR and of vulnerability to future life challenges.

## The Brain Reserve

### Biology

The concept of BR is defined by many biological quantitative (Barulli and Stern, 2013; Mathias and Wheaton, 2015) and qualitative (Mathias and Wheaton, 2015; Jansen et al., 2019) factors. These define a tolerance threshold for a certain degree of injury, after which clinical difficulties can be observed (Barulli and Stern, 2013). A set of variables can be used to measure this threshold (Satz et al., 2011). For instance, the total volume of the brain of individuals has been related to the risk of developing dementia (Mortimer et al., 2003; Groot et al., 2018) so that a lower volume may reflect a lower BR. Similarly, some results indicate that genetic factors could influence and modulate expression of neural pathology (Sleegers et al., 2010; Scheltens et al., 2016; Selkoe and Hardy, 2016).

Presented as a passive model of CR, BR – also known as Threshold Model (Katzman, 1993; Barulli and Stern, 2013) – refers to structural brain properties. In other words, it accounts for inter-individual differences in tolerance to brain injury, especially through the quantitative aspects of the neural substrate (e.g., brain volume, the number of neurons or synapses). This concept also includes a qualitative side (e.g., neurogenesis, brain plasticity). Thus, passive models of CR refer to a set of structural and functional mechanisms to compensate for brain damage in performing a task. Regarding neurodegenerative pathologies, it has been shown that CR can delay the onset of symptoms (Soldan et al., 2020). For an equivalent level of clinical impairment, a patient with a higher CR will have a higher lesion concentration (Scarmeas and Stern, 2003).

Connectivity can also be used to determine this threshold. Indeed, along with other aforementioned factors, network integrity is also associated with the development of functional disorders (Wang et al., 2012; Chao et al., 2013; Medaglia et al., 2017). Thus, it seems that presenting a greater quantity of neurons or synapses, being physically taller or having a greater volume would constitute protective factors regarding the expression of determined brain damage.

On the other hand, the active model of CR, also called by the same name (i.e., CR) or Compensation (Davis et al., 1999), accounts for inter-individual differences in brain network usage when performing the same task (Stern, 2006). Various studies have attempted to define the best indicators of CR, considering measures as varied as education, leisure activities and life experiences. The hypothesis is that all of these experiences would model the cognitive networks and enable individuals to cope with brain pathology more or less successfully (Resende et al., 2018).

## The Cognitive Reserve in Aging Population

### Education

By far the most used proxy in CR literature (Xu et al., 2015), education can be measured in at least two ways: quantitatively (i.e., years of study) or qualitatively (i.e., regarding the quality of education/attainment). More convenient to use, the number of years of study is the most frequently used measure. In healthy subjects, it has been shown that the years of education can modulate functional relationships between brain regions and a given performance (Richards and Deary, 2005; Clouston et al., 2019). Thus, education would allow the use of memory strategies, based on the hippocampal functioning, to enhance performances. Similarly, in mildly impaired individuals, Mungas et al. (2018) have shown that a higher education level could modulate the rate of cognitive decline related to brain atrophy.

Regarding AD, several studies have highlighted a relationship between education and the expression of the disease. Indeed, it has been shown that a lower level of education correlates with a higher risk of AD diagnosis (Meng and D’arcy, 2012). Once the pathology emerges, an effect of the education on the expression of the pathology was emphasized. For example, regarding amyloid lesions, emblematic of AD, it has been shown that the higher the level of education, the higher the lesion load required in order to be expressed clinically (Bennett et al., 2003; Roe et al., 2008; Rentz et al., 2010; Jansen et al., 2018). This effect was also observed, even more strongly, in relation to neurofibrillary tangles (Bennett et al., 2003). Bennett et al. (2003) offer a parallel with studies showing the positive effect of an enriched environment (see section “Environment” below), assuming that education could elicit plasticity in humans, just as environment in animals.

Even within education studies, literacy itself is also a widely used proxy, being considered as very close and effectively reflecting both quantity and quality of education (Xu et al., 2015). For instance, adjusted for age and gender, this factor could predict performance in memory tests (Fyffe et al., 2011). In AD, some studies showed that patients with greater pre-morbid reading capacities demonstrated a faster decline in their cognitive abilities during longitudinal follow-up (Wilson et al., 2000).

The reading activity is based on the integrity and functioning of a distributed brain network, mainly belonging to the left hemisphere (Barquero et al., 2014), but also involving important inter-hemispheric connectivity (Carreiras et al., 2009). Cognitive studies showed morphological and functional differences between the brains of literate and illiterates adults depending on the reading level of the participants (Dehaene, 2018). Similarly, in illiterate adults learning to read, significant brain changes – both structural and functional, and irrespective of overall cognitive efficiency – can be observed as literacy skills are acquired (Carreiras et al., 2009; Dehaene et al., 2010; Dehaene and Cohen, 2011).

In the CR framework, this more rapid decline of AD participants with a pre-morbid level of education and/or higher cognitive functioning is generally explained by the threshold models presented above. The authors postulate that different contingencies throughout life would shape cognitive functioning (Katzman, 1995), inducing higher levels of protection to equivalent levels of brain damage. Once this threshold is exceeded, the participant would present a higher level of impairment, with the pathology evolving in the background over a longer period of time, resulting in a more rapid decline (Satz, 1993; Katzman, 1995).

Overall, it seems that educational attainment, in particular literacy, could act positively on CR, helping patients to cope with brain damages. Nevertheless, although important, education is not an isolated factor in coping abilities, and others, in particular the environment, would influence CR.

### Environment

Studies of pathological mice models have demonstrated the interest of the enriched environment in the expression of lesions in AD. Some studies have shown positive effects on cognitive abilities (Sampedro-Piquero and Begega, 2017; Zhang et al., 2018), especially memory (Jin et al., 2017), and even an effect on lesions themselves (Rodriguez et al., 2013; Klein et al., 2017; Selvi et al., 2017; Zhang et al., 2018). On the contrary, a negative influence of an impoverished environment has also been demonstrated (Volkers and Scherder, 2011). Taking these results as a basis, some authors drew parallels with humans.

Many studies in both animals and humans have demonstrated a significant and positive effect of an enriched environment on the neurogenesis of the hippocampus (Richards and Frankland, 2017). For instance, the results would show a protective effect of cognitive functioning, resulting in a slower decline (Xu et al., 2015). Likewise, in AD, a protection, or even inversion of the deficits has been observed (Xu et al., 2015). Similarly, the extent of the social network has a positive influence on the risk of AD, and can also slow down the cognitive decline (Xu et al., 2015). Older people generally suffer from a social network impoverishment (Freedman and Nicolle, 2020), being one of the most frequent complaints of this population (Miranda-Castillo et al., 2010) and a risk factor (Fratiglioni et al., 2000). Thereby, the introduction of support groups would not only significantly improve the quality of life, but also depressive and behavioral symptoms in mild AD (Nelson and Tabet, 2015). In the same way, the social network density would have a strong role on cognitive functioning in AD (Xu et al., 2015).

Altogether, in both animals and humans, many studies have highlighted the positive effect of an enriched environment on a variety of factors related to cerebral health, including for example the neurogenesis of the hippocampus (Richards and Frankland, 2017). Moreover, besides these external factors, some studies have emphasized the importance of internal ones (i.e., psychological variables).

### Psychological Factors

Many studies have shown an influence of emotions on cognitive performance (Dolan, 2002; Carvalho and Ready, 2010; Brosch et al., 2013). For instance, in dementia, the presence of depression would influence negatively the clinical phenotype (Opdebeeck et al., 2018). It appears that the combination of high (Spitznagel et al., 2006) or low (Opdebeeck et al., 2015) depression and CR is related to symptomatic expression. Similarly, depression is recognized as a significant risk factor in early onset dementia (Barnes and Yaffe, 2011; Byers and Yaffe, 2011; Scheltens et al., 2016; Bos et al., 2017; Planton et al., 2017). Regarding AD, presenting a psychiatric or behavioral syndrome has been shown to accelerate cognitive and functional decline (Nelson and Tabet, 2015).

Overall, it seems that a link can be established between CR on the one hand and psychological factors on the other. The findings indicate that a high CR and absence of psychological disorders would allow better coping with brain pathology. We could also hypothesize that high CR could be protective against psychological harm. Moreover, it has been shown that activity would also both reduce the risk of depression (Cadden et al., 2018; Patel et al., 2018) and contribute positively to CR.

### Activities

As for education, the level of activity can be divided into two modalities with intellectual activity on the one hand, and PA on the other.

Concerning intellectual activity, many authors highlight its protecting role regarding the risk of AD (Barnes and Yaffe, 2011; Scheltens et al., 2016). In adulthood, two variables are considered: occupation and leisure activities. Regarding occupation, it appears that increasing the complexity of activities would be accompanied by a faster cognitive decline when AD occurs, with higher CR being protective by delaying the clinical expression of lesions (Xu et al., 2015; Lee et al., 2019). Some results show a relationship between the amount of brain injury on the one hand and the degree of engagement in intellectual activities on the other hand (Landau et al., 2012). Similarly, leisure seems to follow the same pattern. Indeed, engagement in intellectual activities throughout life seems to have an impact on the rate of decline (in MCI or AD individuals) and on the occurrence of dementia (Xu et al., 2015).

Physical activity would also play a significant role (Barnes and Yaffe, 2011; Baumgart et al., 2015; Dubois et al., 2016; Scheltens et al., 2016) – although weak (Ludyga et al., 2020) – and is considered as one of the most important modifiable factors (Sabia et al., 2017). For a long time, and in particular studies on mice models (Intlekofer and Cotman, 2013), physical exercise has been thought of as neuroprotective for neurodegenerative pathologies (Paillard et al., 2015). PA could thus influence age-related decline (Hamer et al., 2018; Lerche et al., 2018; Loprinzi et al., 2018; Engeroff et al., 2019) – particularly on episodic memory (Hamer et al., 2018) – and would be related to a lower risk of later onset of dementia (Guure et al., 2017; Zotcheva et al., 2018). As for the underlying mechanisms, two modes of action are reported. On the biological level, some results suggest that sports practice would reduce cardiovascular risks (Nelson and Tabet, 2015; Hamer et al., 2018) and/or an increase in the production of neurotrophic factors as well as neuronal excitability (Nelson and Tabet, 2015; Loprinzi et al., 2018). Stimulating the production of new synapses, PA would indirectly have a significant effect on brain plasticity and therefore on memory (Lourenco et al., 2019).

PA has also been quantitatively correlated with the level of *A*β by increasing its clearance rate or reducing its deposition (Intlekofer and Cotman, 2013). In addition, PA would increase brain flow in the region of the hippocampal dentate gyrus with a possible improvement in neurogenesis (Paillard et al., 2015). In healthy elderly humans, some studies have shown an impact of the participants’ sports history on both cognition (Nelson and Tabet, 2015) and brain structure. PA would thereby significantly moderate age-related atrophy in several brain areas, including frontal (Nelson and Tabet, 2015) and medial temporal (especially hippocampal) regions (Intlekofer and Cotman, 2013). Studies involving various types of PA have shown a significant reduction in cognitive decline in AD, despite important methodological variability (Intlekofer and Cotman, 2013; Nelson and Tabet, 2015).

Overall, the findings tend to show that many factors could influence individuals’ abilities to cope with brain damages. Those variables are both internal and external, modifiable and not. In adulthood, the most studied variable seems to be education, which would be an efficient proxy for CR. But less is known about these factors and their effects on individual development.

## Development of Cognitive Reserve

We have seen that the expression of lesions can be modulated by numerous epigenetic factors. Among the environmental conditions, education indeed plays a critical part. However, the majority of these studies focus on adult or even older populations and there are fewer studies on the development of these protective factors during childhood. Considering the work on brain plasticity and sensitive periods during childhood, it can be hypothesized that this time constitutes a decisive period in the emergence and strength of CR (Richards et al., 2013). Stimulation of CR during childhood could thus influence the nature of cognitive aging.

### Childhood Environment

The first years of life are the most determining period in brain development, organization and specialization. Neuronal and volume development grows fourfold between birth and early adulthood (Giedd et al., 1999; Johnson, 2001), enabling optimal action of brain plasticity. On a neurodevelopmental level, recent research has shown that the genetic heritage, the developmental timetable, but also stimulations from surrounding sources and environmental enrichment have a direct impact on the human brain structural networks (Gogtay et al., 2004; Baroncelli et al., 2010; Rao et al., 2010; Anderson et al., 2011; Huang et al., 2013). As a matter of fact, a higher level of stimulation (e.g., parental and environmental demand, learning activities) generates an increased synaptic proliferation, a denser cortex, and a greater intellectual flexibility (Hochberg et al., 2010; Murgatroyd and Spengler, 2011), preparing for reinforcing neuronal resistance to natural aging brain or potential brain damage (Stern, 2006; Asaridou et al., 2020).

Although their association tends to decrease due to a slight improvement in access to education around the world, educational attainment and social origin remain strongly linked (Bernardi and Ballarino, 2016; Mirowsky, 2017). Children from families with higher social class background obtain better scores on achievement tests and better grades in school than children from underprivileged backgrounds (Mullis et al., 2004; Hair et al., 2015). Moreover, children who experienced family persistent poverty perform almost 20 percentile ranks lower than other children at cognitive development test scores, even after controlling for parental investment and others background conditions (Dickerson and Popli, 2016). Similarly, a recent neuroanatomical study tends to show some differences in brain structure of children and adolescents with low or high-income families, particularly in brain regions supporting language, reading, executive function and spatial skills (Kolb and Gibb, 2015; Noble et al., 2015). Studies also linked socioeconomic factors with hippocampal and amygdala volumes (Hanson et al., 2011; Luby et al., 2013). However, other studies report no association (Hanson et al., 2011; Jednoróg et al., 2012). Parental socioeconomic status has therefore a strong impact on children’s cognitive abilities from an early age, and this condition has a direct link to future educational background (Erola et al., 2016). In this regard, research has shown that home environment and adequate parenting (e.g., stimulations, interactions) enhance a better understanding of this link. Parents with higher level of education are better informed about protective or deleterious environmental factors to ensure children’s optimal development, starting from prenatal life (Prickett and Augustine, 2016; Jeong et al., 2018). Studies have also highlighted that time investment from parents on children is linked to parental economic level (Park, 2008) and has a major impact on cognitive outcomes in childhood (Cunha and Heckman, 2009; Del Boca et al., 2017). This investment generally involves the amount of time children spend in physical or cultural activities with their father, mother, or both, but also their general exposure to discussion and social interaction. However, a further study shows that a child’s and adolescent’s own investment also matters, defeating an immutable social determinism (Del Boca et al., 2017). Moreover, level of interest in books and arts is significantly higher before 12 years old and seems not related to parental income status (Nanhou et al., 2016). Nevertheless, growing up in advantaged socioeconomic conditions and a stimulating environment in childhood seems to facilitate CR development (Stern, 2009; Aartsen et al., 2019).

### Psychosocial and Psycho-Affective Development

The earlier stages of child development are reported to be deeply self-centered (Heo et al., 2011), although children are already able to show affection and altruistic intentions. Thus, before well-developed language, a child’s learning process is based on imitations, which require interaction with others. This process leads to duplication and strengthening of connections between mirror neurons (Rizzolatti, 2005; Cattaneo and Rizzolatti, 2009). This growing network provides empathetic movements (i.e., affective theory of mind) (Kaplan and Iacoboni, 2006). It also allows better abilities to understand interpersonal relationships (e.g., connivance, conflicts) and others’ mental states (i.e., based on beliefs, intentions, feelings…), aims and achievements (i.e., cognitive theory of mind), for more nuanced language processing. Development of social cognition, through both sides of theory of mind, helps children to progressively decrease their self-centered perception by prosocial behaviors and to develop and balance their emotional regulation.

This balance allows social adaptation and individual well-being (Cole et al., 2004; Spinrad et al., 2006), such that proper socio-cognitive abilities contribute to empathetic and prosocial behaviors. They provide help to adapt feelings and emotional expression according to the context, and to solve some negative or disturbing situations through appropriate strategies. As a result, a well-balanced emotional regulation contributes to reduced psychopathological risk factors.

From a cognitive perspective, those socio-cognitive abilities are also interrelated with the learning capacity of children using motivation and executive functions (Rueda et al., 2004; Posner et al., 2007), future language skills through early sensory experiences (e.g., eye tracking, facial emotional expression, sensorial communication), frequency and levels of communication (Rosenzweig, 2003; Cates et al., 2012). The interest in physical and emotional experimentation with the surrounding world increases, as well as the focus abilities with sustained attention.

Furthermore, reading abilities develop specific areas in visual cortex, contributing to decrypt shapes and face recognition (Rueckl et al., 2015; Dehaene, 2018), and allowing faster processing of visual information into sounds and meanings (Bouhali et al., 2014; Saygin et al., 2016). As an extension, research underlines a close relationship between reading skills, emotional distress and behavior issues. Studies and meta-analysis show that about 60% of kids in school with emotional and behavioral troubles have difficulties with basic reading and comprehension. Interestingly regarding adult outcomes, those troubles tend to remain with time (Kauffman, 2001; Nelson et al., 2003). Thus, access to early childhood education is a determinant factor in cognitive and psychosocial development.

### Childhood Education

Low linguistic ability in early life is a strong predictor of poor cognitive function and AD in late life (Lo et al., 2013; Deckers et al., 2014). Research shows that literacy learning and reading skills are identified as critical prerequisites of cognition and socio-emotional development in childhood (Wehbe et al., 2014).

The learning of reading in children is thus accompanied by progressive brain changes (Dehaene-Lambertz et al., 2018). In group studies, children with or without reading difficulties show differences in activity of certain regions of the left hemisphere, including a lesser activation of temporo-parietal (Barquero et al., 2014) and occipito-temporal areas, as well as hyper activation of the inferior frontal gyrus (Barquero et al., 2014; Dehaene-Lambertz et al., 2018). These differences would persist in adults with reading difficulties. Compensatory hyper activation would also be demonstrated in the right hemisphere.

#### On Attention

Interacting with a book while listening to a story is the backbone of joint attention, which is a basic function of future attention skills, double task processing, empathetic behaviors and eye gaze meaning (Girardot et al., 2009). A 3-year longitudinal study shows that attentional skills, and particularly visual attention in preschoolers, predict quality of future reading acquisition (Franceschini et al., 2012). The same longitudinal study reports that about 60% of poor readers at school displayed visual-attention deficit in preschool.

#### On Mathematical Achievements

An early reading exposure program for children from low-income families showed better academic results and cognitive abilities (particularly in mathematics), with long-term effects, as compared with children from the same environment who have not followed the program (Campbell et al., 2001). Thus, mathematical reasoning is directly related to adequate early reading exposure, in particular with regard to phonological awareness and syntax (Gathercole et al., 2006; Chow and Ekholm, 2019).

#### On Intelligence

Studies show that children with an early psychosocial stimulation (e.g., play session program or assessment on play time at home with parent-children interaction) also have a higher adult IQ and better general knowledge, as compared to peers without such stimulation (Raine et al., 2002; Walker et al., 2011). Raine’s longitudinal study shows that increased stimulation seeking at 3 years old enhanced both scholastic performance and neuropsychological results in later childhood (i.e., at age 11) (Raine et al., 2002). This link is not mediated by parental education and occupation. A common hypothesis is that early cognitive stimulation creates a continuous enrichment of the environment that stimulates brain development. Moreover, this study suggests that a sensation-seeking personality trait in children is associated with more PA, and that this PA may be more impactful on increased cognitive ability.

#### On Working Memory and Non-verbal Reasoning

Studies show that working memory is a prerequisite of skill and knowledge in reading comprehension. Working memory deficit is moreover a very well-known diagnostic criterion for dyslexia (Gathercole et al., 2006; McVay and Kane, 2012; American Psychiatric Association, 2013; Wu et al., 2017). Conversely, early reading abilities extend the working memory process. An experiment of exposure to books and initiation to manual arts has been done in 49 children, aged from 7 to 12 (Wandell et al., 2008). Over 2 years, significant impact on intellectual abilities (notably on processing speed) and on reading skills were observed. From all activities, reading is the most stimulating on phonological awareness, hearing and attention span/memorizing activities. Lastly, exposure to reading and listening to stories between 3 and 5 years old may promote activation of cerebral areas in charge of mental imagery, narrative understanding, memory and imagination (Hutton et al., 2015). Those abilities are also stimulated in a different but complementary way by extracurricular activities.

### Childhood Activities

According to some studies, artistic and play stimulation significantly increases the capacity for sustained attention (Wolfe and Noguchi, 2009), including motivation sources (Kieras, 2006) through feedback. At the age of 6 years old, children who learn musical instruments are more impervious to noise (i.e., more resistant to auditory distraction) (Strait and Kraus, 2011). This improvement has a significant impact on attention and intellectual efficiency test scores (Posner and Patoine, 2009). The access to exhibition or initiation in visual arts, music and dance over 2 years significantly improves intellectual efficiency (especially the processing speed) and reading skills (Wandell et al., 2008). But music is the most effective art for working memory and reading abilities (i.e., phonological awareness, hearing). Children receiving musical education also have denser neural connections in the areas governing verbal communication (Moreno et al., 2011). Moreover, functional magnetic resonance imaging (fMRI) studies show common brain activations during musical learning and mathematical thinking. To assess the impact of artistic stimulation on scholastic performance, Wandell et al. (2008) showed that learning a musical instrument improved mathematics (+50%) and history-geography (+40%) grades in children from underprivileged environments (study on 300,000 American students). Music also increases recall in memory and visual imagery. Overall, learning to play an instrument also promotes some motor and coordination skills, creating deep and permanent changes in the brain (Schlaug et al., 2005).

In line with artistic activities, children’s “free play” also develops language, emotional regulation, imagination and helps to densify the socio-behavioral responses to obstacles and dilemmas (Lillard et al., 2013). Thus, free play allows a reinforcement of the identification (i.e., by the appropriation of other identities) and increases self-esteem. But free play requires some free time and some space which are not available in all families. Indeed, since the 2000s, the use of digital technologies (e.g., video games, internet use) has been an integral part of children’s activities (screen time has been estimated to be around 8 h a week for 3-4-year-olds in the United Kingdom) (Kabali et al., 2015; Children’s Commissioner, 2017). The debate around children’s digital activities covers many aspects: the potential consequences on the brain development of toddlers (Johnson, 2015; Reid Chassiakos et al., 2016), the risks related to children’s exposure to inappropriate images (Tomopoulos et al., 2010), and the unpermitted use of a child’s image [risks that have been very well covered by Sziron and Hildt (2018)], to name but a few. From a cognitive developmental perspective, some research considers that access to screens facilitates the stimulation of communication, literacy or executive function development (Courage and Setliff, 2010; Neumann, 2016; Huber et al., 2018), while others consider that it diminishes experiences, quality of interpersonal relationships (i.e., communication, empathy), outdoor activities, healthy living (i.e., well-being, sleep and food) (Zimmerman et al., 2007; Lin et al., 2015; Kirkorian, 2018; Stiglic and Viner, 2019).

Without settling this debate here, an emerging literature seems, however, to observe that screen time, being done at the expense of other activities and real stimulations, contributes to weakening children’s cognitive development (e.g., language, attention skills, behavior regulation) (Christakis, 2009). Moreover, excessive screen use is more at risk in low-income families (Atkin et al., 2014; Hinkley et al., 2014). In this context, a drastic limitation of the use of digital technologies, as well as restriction to content that is compatible with developmental capacities, remains advocated in clinical practice (American Academy of Pediatrics, 2013). Based on the hypotheses defended in this article and informed by the literature described above, we could therefore consider that the effects of the massive and unsupervised use of new technologies at an early age can undermine the capitalization of a robust CR. Conversely, a wise and intentioned use of adapted and age-targeted content can accompany its reinforcement.

**FIGURE 1:**
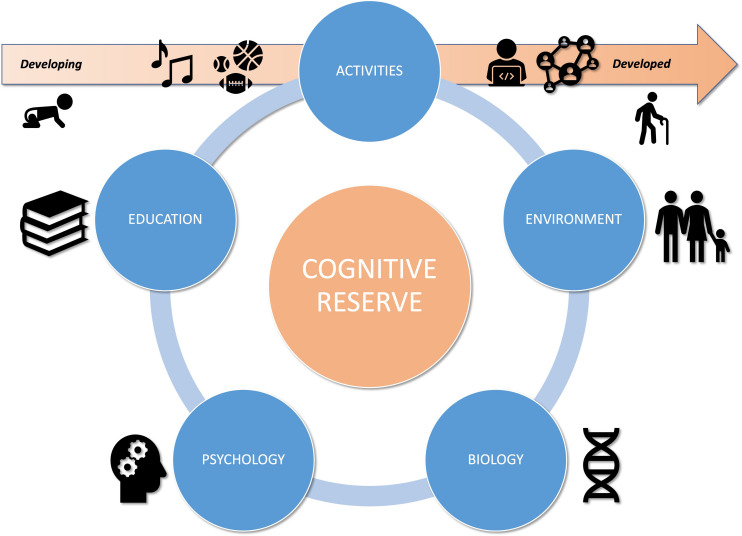
Title: CR factors dynamic interactions in a lifespan perspective.

To sum up, cognitive and psycho-social development are interlinked but also heterogeneous, mostly because of environmental inequalities: parental socio-economic and socio-cultural status, family stress and behavioral patterns (e.g., nutrition, sleep, screen-time) (Cantin et al., 2012; Duncan and Magnuson, 2012; Noble et al., 2015; Sampedro-Piquero and Begega, 2017), parenting style (e.g., education, level of stimulation, usage, attitude, attachment to school, reading level and interest in books) (Del Boca et al., 2017), methods and approach to learning (e.g., Montessori pedagogy, Public/Private school…) (Al-Mansour and Al-Shorman, 2011; Dehaene, 2018), and access to extracurricular activities (Nanhou et al., 2016) (see Figure 1).

## Discussion

In view of these studies, early stimulations with human sensory interactions, language, music and play help children to develop robust and flexible cognitive, affective and psychosocial abilities. By confronting data from adult studies, we may venture the hypothesis that CR origin and development take place beginning in early childhood. We could consider childhood as the first act of primary prevention of pathological aging and loss of cognitive abilities. Indeed, densifying synaptic reserve and increasing CR as early as possible, and then maintaining and strengthening it throughout adulthood, could contribute to giving people, caregivers and health professionals the skills to live a better, happier life and to postpone dementia.

This association made between the developing and the developed CR is therefore currently theoretical and based on an intellectual construction, but could become tomorrow scientifically attested. In order to follow the impact of these links, we could imagine the creation of a longitudinal epidemiological database. It could collect environmental and socio-cognitive data from children and monitor their progress, through indicators on their education, job, quality of life and health until their end of life.

By supporting this hypothesis, early environmental stimulation would therefore be seen as a true primary prevention and a public health matter. Identifying neurodevelopmental disorders, which can affect personal and professional outcomes (Johnson and Reader, 2002; Mannuzza et al., 2004; Alexander-Passe, 2006; Barkley et al., 2006; Retz and Rösler, 2009; Daley and Birchwood, 2010; Brod et al., 2012; Lichtenstein et al., 2012; Wymbs et al., 2012; Groenman et al., 2013; Gaïffas et al., 2014; Harpin et al., 2016; Schoentgen, 2017), would therefore have much longer-term impact. In addition, this research would motivate the development of innovative school pedagogies, especially for children with academic difficulties. Finally, considering these developmental elements could provide benefits in aging and, by delaying and reducing the risk of their occurrence, have a positive impact on the treatment of neurodegenerative pathologies.

## Author Contributions

BS and GG contributed equally to the bibliography research and writing the manuscript. BD supervised conceptual orientation of this manuscript. All authors contributed to the article and approved the submitted version.

## Conflict of Interest

The authors declare that the research was conducted in the absence of any commercial or financial relationships that could be construed as a potential conflict of interest.

## Abbreviations

AD: Alzheimer’s disease
BR: brain reserve
CR: cognitive reserve
MCI: mild cognitive impairment
MRI: magnetic resonance imaging
PA: physical activity
WHO: world health organization.
